# Religiosity and Suicidal Ideation in Patients With Depression

**DOI:** 10.7759/cureus.85826

**Published:** 2025-06-12

**Authors:** Sachin U Ghatge, Bibaswan Mallik, Anand M Anuse

**Affiliations:** 1 Psychiatry, Bharati Vidyapeeth (Deemed to be University) Medical College and Hospital, Sangli, Sangli, IND

**Keywords:** depression, religiosity, spirituality, suicidal ideation, suicidality

## Abstract

Background: The exact role of religiosity in mental health disorders is much debated, particularly in the context of a diverse country such as India.

Aim: To study the religiosity and suicidal ideation in patients with depression at a tertiary care hospital in Southern Maharashtra in the year 2024.

Materials and methods: Forty consecutively treated patients with depression, above the age of 18 years, in a tertiary care institute were recruited. Subjects were administered a semi-structured proforma, along with the Duke University Religious Index (DUREL) and the Scale for Suicidal Ideation. Data were collected and compiled. Statistical analysis was carried out using Microsoft Excel 365 (Microsoft® Corp., Redmond, WA) and Statistical Product and Service Solutions (SPSS, version 29; IBM SPSS Statistics for Windows, Armonk, NY).

Results: There is a statistically significant difference between the mean DUREL scores and the scale for suicidal ideation (SSI) classifications as per the Mann-Whitney U test.

Conclusion: There is an inverse correlation between religiosity, as quantified by the DUREL, and suicidal ideation.

## Introduction

Religiosity can be defined as “the self-perceived importance of religion and the degree to which religious beliefs and identities translate into secular attitudes” [1-3]. Religiosity consists of elements associated with belonging to a group of people who have similar beliefs and pursuits. Spirituality can be understood as an element that exists both inside and outside of a religious setting and is defined by a desire for transcendence, a sense of connection, and a purpose with life [4]. Given that a large percentage of people exhibit feelings of uneasiness and uncertainty regarding the present and the future, religiosity may have a substantial impact on the mental health of most individuals [1]. Both spirituality and religion can provide a person with a strong sense of meaning in life, lessen stress, and offer coping techniques [5]. Approximately 27% of people identify as just spiritual, despite the fact that many people may identify as both religious and spiritual, indicating that these conceptions are distinct [6]. An increasing amount of research suggests that the idea of self-control may contribute to the explanation of the religion-health relationship [7]. Evidence suggests that health teams are uneasy and unprepared to deal with religiosity as an expression of spirituality, despite the fact that religiosity has been shown to have a positive impact on the lives of people with mental illnesses. Since this is one of the dimensions of health, the existence of this gap in the comprehensive care strategy can be deduced from the results [2]. Poor relationship quality, smoking, low self-esteem, functional impairment from a disability or illness, and a history of depression in one's family or lifetime are risk factors for depression in midlife [8]. A small number of studies have shown that religiosity is linked to midlife mental health and protects against depression [9]. Suicidal thoughts and actions are thought to be more likely among people who suffer from major depression [10]. Depression, suicidal thoughts and actions, and drug abuse are generally negatively correlated with one's level of religious participation [11]. According to certain research, religious habits and impulsivity are inversely correlated [12]. It was shown that the characteristics of universal religion, forgiving others, God as judge, and involved God were linked to a lower incidence of externalizing disorders, such as adult antisocial conduct and substance dependence on alcohol, cigarettes, and other drugs [13]. Studies have shown that children's physical and mental outcomes are positively correlated with their parents' religiosity [14].

Hence, as is evident from the above discussion, there is a role of religiosity in the aspects of impulsivity and suicidality, but the exact role is not agreed upon. The aim of our research is to study the religiosity and suicidal ideation in patients with depression at a tertiary care hospital in Southern Maharashtra in the year 2024. We hope that the findings will aid in framing policy with regard to assessment, treatment, and therapy in patients with depression.

## Materials and methods

The study was conducted in the Psychiatry Department of a Bharati Vidyapeeth (Deemed to be University) Medical College and Hospital, Sangli, India, after permission was taken from the Head of Department. It was approved by the Institutional Ethics Committee (via letter number 576/24), for a time period of 173 days (five months and 20 days).

Subjects: We recruited patients above the age of 18, meeting the criteria for depressive episode, seeking treatment at a tertiary care hospital in Maharashtra, and not meeting the diagnostic criteria for organic mood disorder, mental retardation, chronic renal failure, cancer, chronic liver disease, congestive heart failure, substance intoxication, substance withdrawal, or substance abuse. The study was approved by the Institutional Ethics Review Board. Convenience sampling techniques were used for the enrolment of subjects into the study. A total of 40 subjects were enrolled in the study.

Screening: Subjects diagnosed to have depressive episode, recurrent depressive disorder, bipolar affective disorder, and current depressive episode as per the International Classification of Diseases, Tenth Revision (ICD-10) [15] criteria for research were included in the study. Subjects below the age of 18 were excluded from the study, as were those subjects not willing to give consent to participate in the study.

Study type: This is a hospital-based, cross-sectional study.

Assessment: The subjects were then administered a semi-structured proforma, via which the sociodemographic details (age, gender, education level, residence type, marital status) and clinical details (diagnosis, duration of illness, family history of suicide, medical comorbidity) were collected.

The subjects were also administered the scale for suicidal ideation (SSI), which was designed to measure the intensity, pervasiveness, and characteristics of suicidal ideation in adults and to assess the risk of later suicide attempts in individuals who have thoughts, plans, and wishes to commit suicide. It is a well-established clinician rating scale and is presented in a semi-structured interview format. It has high validity and reliability values (validity value ranging between 0.50 and 0.75, and a Cronbach’s alpha value of 0.84-0.9). The scale has 18 items, each scored from zero to three, with a maximum score of 54. Suicidality is classified based on the scores attained, with scores between 0 and 8 indicating mild suicidal ideation, scores between 9 and 20 indicating moderate suicidal ideation, and scores between 21 and 54 indicating severe suicidal ideation [16].

Religiosity was assessed using the Duke University Religion Index (DUREL), which was found to be a reliable and valid measure of religiosity. The two-week test-retest reliability of the DUREL is high (validity value ranging from 0.79 to 0.86, and a Cronbach's alpha value of 0.91). It is a well-established clinician rating scale and is presented in a semi-structured interview format. The scale consists of two items scored from 1 to 6, and three items scored from 1 to 5, with a total maximal score of 27. There are no universally accepted classifications of DUREL scores. For the purposes of convenience, the scores were put into groups, namely, scores between 5 and 11, scores between 12 and 17, scores between 18 and 24, and scores between 25 and 27 [17].

Statistical analysis: Statistical analysis was done using Microsoft Excel 365 (Microsoft® Corp., Redmond, WA) and Statistical Product and Service Solutions (SPSS, version 29; IBM SPSS Statistics for Windows, Armonk, NY). Frequency and percentages were obtained for qualitative characters. The mean and standard deviation were calculated for the SSI score and DUREL score using the Mann-Whitney U test and independent sample t-test.

## Results

Forty participants were recruited in this study at the time of their diagnosis, before the initiation of treatment. All the participants were diagnosed cases of depressive episodes.

In Table 1, we provide a statistical overview of the sociodemographic details of our study subjects. Of the 40 subjects, 19 (47.5%) were in the age group of 18-34 years, 17 (42.5%) belonged to the age group of 35-50 years, three (7.5%) belonged to the age group 51-65, and one (2.5%) belonged to the age group of 65 years and above. Thirteen of our subjects are males (32.5%), and 27 (67.5%) are females. Four of the participants were primary educated (10%), while 36 (90%) had higher education. Thirteen had a rural residence (32.5%), and 27 had an urban residence (67.5%). Twenty-eight of the subjects were married (70%), and 12 were unmarried (30%).

**Table 1:** Frequency and percentage of sociodemographic parameters of the study participants

Parameters		Frequency	Percentage
Age	18-34	19	47.5
	35-50	17	42.5
	51-65	3	7.5
	65+	1	2.5
Gender	Male	13	32.5
	Female	27	67.5
Education	Primary	4	10
	Higher	36	90
Residence	Urban	13	32.5
	Rural	27	67.5
Marital Status	Married	28	70
	Unmarried	12	30

In Table 2, we provide a statistical overview of the clinical details of our study subjects. Of the 40 participants, five (12.5%) had a diagnosis of the first episode of depression, 19 (47.5%) had a diagnosis of recurrent depressive disorder, and 16 (40%) had a diagnosis of bipolar affective disorder. Four had an illness duration of less than one month (10%), one had an illness duration of one to six months (2.5%), 14 had an illness duration of one to five years (35%), and 21 (52.5%) had an illness duration more than five years. Five of the subjects had a family history of completed suicide (12.5%), and 35 had no family history of completed suicide (87.5%). Thirty-one (77.5%) had no medical comorbidity, six had a history of hypertension (15%), two had diabetes mellitus (5%), and one had a history of thyroid disorder (2.5%).

**Table 2:** Frequency and percentage of clinical parameters of the study participants

Parameters		Frequency	Percentage
Diagnosis	Depressive Episode	5	12.5
	Recurrent Depressive Disorder	19	47.5
	Bipolar Affective Disorder	16	40
Duration of Illness	< 6 Months	4	10
	6 Months-1 Year	1	2.5
	1-5 Years	14	35
	>5 Years	21	52.5
Family History of Completed Suicide	Yes	5	12.5
	No	35	87.5
Comorbidity	Diabetes Mellitus	2	5
	Hypertension	6	15
	Thyroid Disorders	1	2.5
	None	31	77.5

In Table 3, we present a statistical overview with regards to the data of DUREL [17] scores and suicidal ideation of our study subjects. Of the 40 participants, 17 (42.5%) had suicidal ideation, with a mean DUREL [17] score of 16.882, while 23 (57.5%) did not have suicidal ideation, with a mean DUREL [17] score of 18.087. The difference in DUREL [17] scores between the two groups was found to be statistically significant.

**Table 3:** Frequency of the study subjects according to DUREL scores and suicidal ideation DUREL: Duke University Religious Index This is the Mann-Whitney U test.

Variable	Suicidal Ideation	Number of Participants	Mean DUREL Score	P-value
DUREL Score	With Suicidal Ideation	17	16.882	0.028
	Without Suicidal Ideation	23	18.087	

In Table 4, we provide the data regarding DUREL [17] scores and SSI [16] scores of our study subjects. There were two groups among the study subjects, namely, those with DUREL [17] scores between 12 and 17 and those with DUREL [17] scores between 18 and 24, which were then compared to the SSI [16] scores. For the group with DUREL [17] scores between 12 and 17, there is a statistical correlation with the SSI [16] scores, while for those with scores between 18 and 24, no significant correlation was found with SSI [16] scores.

**Table 4:** Frequency of the study subjects according to DUREL and SSI scores DUREL: Duke University Religious Index; SSI: scale for suicidal ideation This is the independent sample t-test value.

Parameters		0-8 Mild Suicidal Ideation	9-20 Moderate Suicidal Ideation	21 and Above Severe Suicidal Ideation	P-value
DUREL Score	12-17	2	14	4	0.021
	18-24	17	3	0	0.635

## Discussion

Religiosity by itself has a varied relevance to mental health and coping, with both protection and causality being described in the literature. Religiosity is generally thought to be a protective factor against suicidal tendencies, owing to the ingrained belief system and concepts of eternal damnation and punishment even after death [5]. However, religious beliefs may add to perceived stress, which is contributory to suicidal ideation [5].

None of the sociodemographic or clinical variables was found to have any bearing on suicidal ideation. We found that higher religiosity scores are not directly correlated with decreased suicidal ideation [18-20]. Lower scores are more directly linked to higher suicidal ideation [13,18,21-24].

The findings in the study are in stark contrast to studies done by Stolz et al. [25] and Tae et al. [26] in that sociodemographic factors were found to have no direct correlation to suicidality in the study subjects. One of the reasons that could be considered is the relatively homogenous belief systems in the populations studied in the studies mentioned above. The Indian population by itself is to be considered to have much variance within those belonging to the same faith system. Additionally, multiple faith systems represent significant portions of the population.

The basic understanding is that religiosity has a positive influence on coping, as is evidenced in studies by Dua et al. [18], Stroppa et al. [23], and Lopez et al. [27]. Better coping, in return, has a protective influence against suicidality [13,18,19,28-30].

The limitations were the lower sample size and study duration. No investigation was carried out with respect to the evaluation of coping skills, the severity of depressive episodes, and the presence of concurrent psychiatric diagnoses, or social factors such as mass media, available material on suicide [31,32]. Further, no evaluation was done to evaluate the role of religious affiliation in suicidality. Treatment history and current ongoing treatment protocols were not taken into account as factors affecting spirituality or suicidality.

The study's strengths are as follows: The data correlating religiosity to suicidality are sparse and conflicting at the same time, particularly in an Indian context, and this study hopes to add to the existing literature. The study population was a mix of outpatient and inpatient treatment patients, and the scales applied for religiosity were based on objective data. Overall, these factors help in supporting the findings of the current research effort, which is also a modest effort in filling the lacunae in the existing literature [33-38].

## Conclusions

Using more sophisticated techniques and general hospital samples, we have observed that, while lower religiosity scores were associated with higher suicidality, no such correlation could be established between higher religiosity scores, which may suggest the presence of a plateau phenomenon after a certain level of religiosity, which also accounts for the conflicting views on the effects of religiosity on coping and suicidality. Such a phenomenon has not been demonstrated in any prior study, with most studies showing either no correlation or association or showing a frank direct or indirect correlation. Further research and investigation are needed to find the exact correlation between religiosity and suicidality, particularly in a diverse country as India.

## Appendices

**Table 5:** Patient proforma

Date	Age	Gender	Education
Patient Code	Marital Status	Residence	
Brief Clinical History			
Diagnosis	Duration of Illness	Comorbidity	Family History of Completed Suicide: Yes/No
Duke University Religious Index Score			
Scale for Suicidal Ideation Score	

**Table 6:** The Duke University Religion Index (DUREL) The DUREL score/index is under the Creative Commons License: https://www.mdpi.com/2077-1444/1/1/78

(1) How often do you attend church or other religious meetings? (ORA)
1 - Never; 2 - Once a year or less; 3 - A few times a year; 4 - A few times a month; 5 - Once a week; 6 - More than once/week
(2) How often do you spend time in private religious activities, such as prayer, meditation or Bible study? (NORA)
1 - Rarely or never; 2 - A few times a month; 3 - Once a week; 4 - Two or more times/week; 5 - Daily; 6 - More than once a day
The following section contains 3 statements about religious belief or experience. Please mark the extent to which each statement is true or not true for you.
(3) In my life, I experience the presence of the Divine (i.e., God) - (IR)
1 - Definitely not true; 2 - Tends not to be true; 3 - Unsure; 4 - Tends to be true; 5 - Definitely true of me
(4) My religious beliefs are what really lie behind my whole approach to life - (IR)
1 - Definitely not true; 2 - Tends not to be true; 3 - Unsure; 4 - Tends to be true; 5 - Definitely true of me
(5) I try hard to carry my religion over into all other dealings in life - (IR)
1 - Definitely not true; 2 - Tends not to be true; 3 - Unsure; 4 - Tends to be true; 5 - Definitely true of me

**Table 7:** Scoring for the Modified Scale for Suicide Ideation (MSSI) scale Permission to use the Modified Scale for Suicide Ideation (MSSI) scale has been granted by the owner Ivan W. Miller.

Item Number	Item Content
1	Wish to die
2	Wish to live
3	Desire - active attempt
4	Desire - passive attempt
5	Duration of thoughts
6	Frequency
7	Intensity
8	Deterrent
9	Reasons
10	Method - specificity
11	Method - availability
12	Courage
13	Competence
14	Expectancy of attempt
15	Talk of death
16	Writing of death
17	Suicide attempt
18	Actual preparation
MSSI TOTAL SCORE:	________________
Severity Categories based on the MSSI Total Score	
0-8 = Low Suicidal Ideation	
9-20 = Mild-Moderate Suicidal Ideation	
21+ = Severe Suicidal Ideation	
